# The complete chloroplast genome of *Hovenia dulcis* (Rhamnaceae)

**DOI:** 10.1080/23802359.2020.1711822

**Published:** 2020-01-16

**Authors:** Li-Zhen Ling, Shu-Dong Zhang

**Affiliations:** School of Biological Sciences and Technology, Liupanshui Normal University, Liupanshui, China

**Keywords:** Chloroplast genome, *Hovenia dulcis*, phylogenetic analysis, Rhamnaceae

## Abstract

The first complete chloroplast (cp) genome of *Hovenia dulcis* was reported in this study. The *H. dulcis* cp genome was 161,636 bp long with two inverted repeat (IR) regions of 26,574 bp, the large single-copy (LSC) region of 89,574 bp, and the small single-copy (SSC) region of 18,914 bp. The cp genome of this species contained 113 genes, including 79 protein-coding genes, 4 ribosomal RNA genes, and 30 transfer RNA genes. The overall GC content was 36.6%. Phylogenetic analysis based on the complete cp genomes within the Rhamnaceae family suggests that *H. dulcis* is closer to the genus of *Ziziphus*.

*Hovenia dulcis* Thunb. is a perennial tree of the family Rhamnaceae. It is commonly found in China, Japan, and Korea. This species has a long history as a food supplement and the main edible parts are the peduncles (Hyun et al. 2010). In East Asia, *H. dulcis* has been used in traditional herbal medicine for the treatment of liver diseases and detoxification after alcoholic poisoning (An et al. 1999; Lim et al. 2016). Recent pharmaceutical studies have shown that the extracts of the fruits, seeds, and branches of *H. dulcis* attenuate acute liver toxicity and atopic dermatitis-like skin lesions and exert antitumor, anti-lipid peroxidation, antisteatotic, anti-inflammatory, antioxidant, and antiallergic activities (Lim et al. 2015; 2016; Choi et al. 2017; Yang et al. 2019). Here, we characterized the complete chloroplast (cp) genome of *H. dulcis* based on the Illumina sequencing technology to understand its genetic background and to explore its phylogenetic placement within Rhamnaceae.

The specimens (lpssy0298) of *H. dulcis* were collected from Qingdao (Shandong, China, N36°08′19″, E120°39′23″, 105 m) and deposited in the herbarium of the Liupanshui Normal University (LPSNU). The total DNA was extracted and used for sequencing as previously described (Zhang et al. 2019). The generated 2 Gb raw data were used for *de novo* cp genome assembly with SPAdes (Bankevich et al. 2012) and all predicted genes were annotated using PGA (Qu et al. 2019). The complete cp genome sequence of *H. dulcis* was deposited in the GenBank database under the accession number MN723868.

The complete cp genome of *H. dulcis* is 161,636 bp in length and shows the GC content of 36.6%. The cp genome of this species displays a typical quadripartite structure, two copies of inverted repeats (IRs, 26,574 bp each) segregated by a large single copy (LSC, 89,574 bp) region and a small single copy (SSC, 18,914 bp) region. In addition, a total of 113 unique genes were encoded, including 79 protein-coding genes (PCGs), 30 transfer RNA (tRNA) genes, and 4 ribosomal RNA (rRNA) genes. Of them, seven PCGs (*ndhB*, *rpl2*, *rpl23*, *rps12*, *rps7*, *ycf15,* and *ycf2*), four rRNAs (*rrn16*, *rrn23*, *rrn4.5,* and *rrn5*), and seven tRNAs (*trnA*-*UGC*, *trnI*-*CAU*, *trnI*-*GAU*, *trnL*-*CAA*, *trnN*-*GUU, trnR-ACG,* and *trnV*-*GAC*) have two copies. Fifteen genes (*atpF*, *ndhA*, *ndhB*, *petB*, *petD*, *rpl16*, *rpl2*, *rpoC1*, *rps16*, *trnA-UGC*, *trnG-UCC*, *trnI-GAU*, *trnK-UUU*, *trnL-UAA,* and *trnV-UAC*) contain one intron and three genes (*clpP*, *rps12,* and *ycf3*) have two introns.

To determine the phylogenetic position of *H. dulcis*, phylogenomic analyses were carried out with the maximum likelihood (ML) and Bayesian inference (BI) methods (Ronquist et al. 2012; Stamatakis 2014). Seven species (including subspecies) from Elaeagnaceae (*Hippophae rhamnoides*, *H. rhamnoides* subsp. *yunnanensis*, *H. gyantsensis*, *Elaeagnus macrophylla*, *E. loureirii*, *E. mollis,* and *E. angustifolia*) were used as outgroups. The cp genomes of *H. dulcis* and previously published species from the Rhamnaceae family were used for phylogenetic analyses. The ML and BI analyses generated the same tree topology (Figure 1). The phylogenetic tree showed that *H. dulcis* is more closely related to the genus *Ziziphus*.

**Figure 1.**
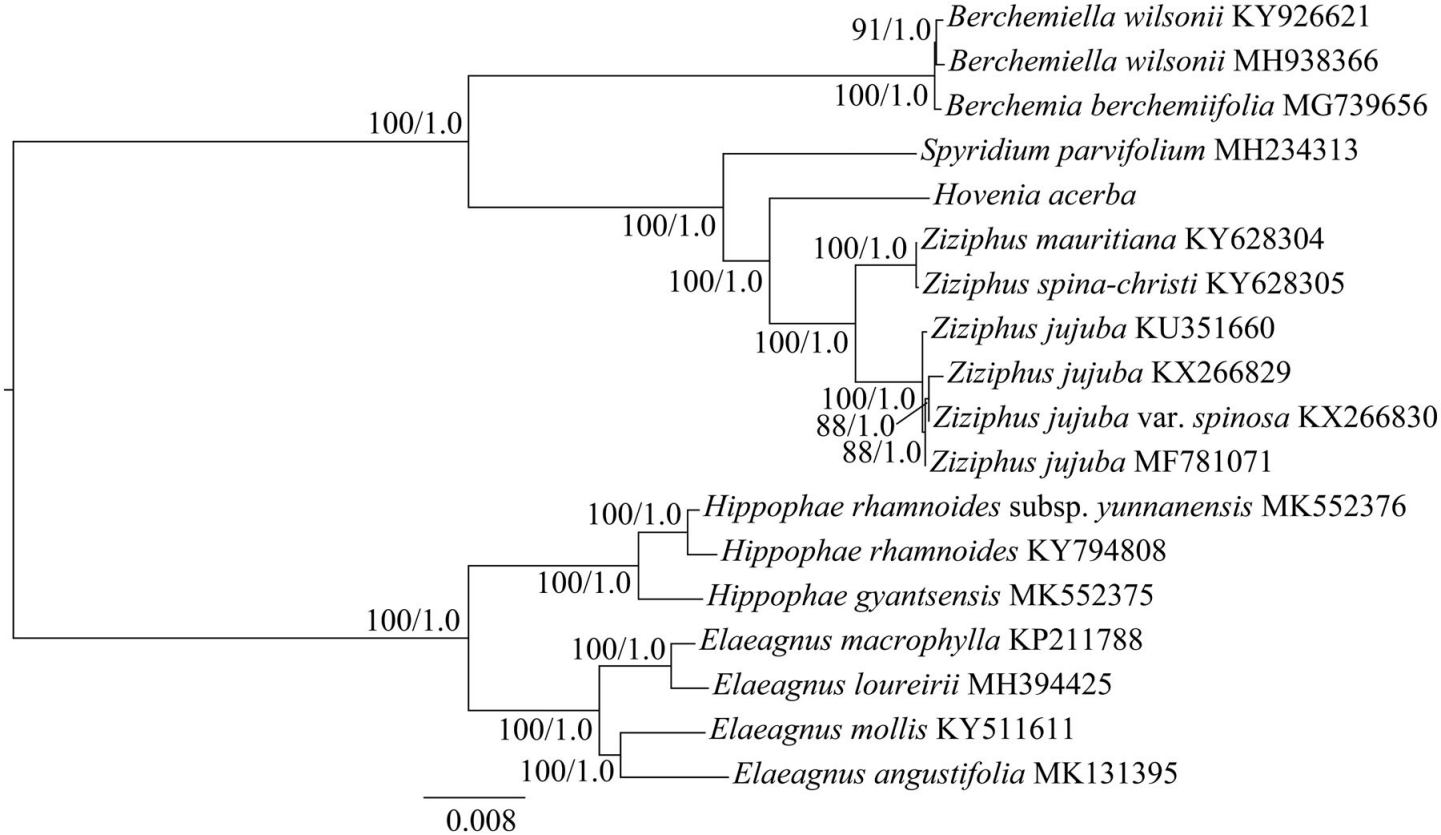
The maximum likelihood (ML) tree of Rhamnaceae inferred from the complete chloroplast genome sequences. Numbers at nodes correspond to ML bootstrap percentages (1,000 replicates) and Bayesian inference (BI) posterior probabilities.
